# Fiber-Optic Pyrometer with Optically Powered Switch for Temperature Measurements

**DOI:** 10.3390/s18020483

**Published:** 2018-02-06

**Authors:** Carmen Vázquez, Sandra Pérez-Prieto, Juan D. López-Cardona, Alberto Tapetado, Enrique Blanco, Jorge Moreno-López, David S. Montero, Pedro C. Lallana

**Affiliations:** Electronics Technology Department, University Carlos III of Madrid, 28911 Leganés, Spain; saperezp@ing.uc3m.es (S.P.-P.); julopezc@ing.uc3m.es (J.D.L.-C.); atapetad@ing.uc3m.es (A.T.); 100303714@alumnos.uc3m.es (E.B.); jorgmore@ing.uc3m.es (J.M.-L.); dsmontero@ing.uc3m.es (D.S.M.); pcontrer@ing.uc3m.es (P.C.L.)

**Keywords:** contactless temperature sensor, fiber-optic, pyrometer, optical switch, power over fiber, optical chopper, lock-in amplifier

## Abstract

We report the experimental results on a new infrared fiber-optic pyrometer for very localized and high-speed temperature measurements ranging from 170 to 530 °C using low-noise photodetectors and high-gain transimpedance amplifiers with a single gain mode in the whole temperature range. We also report a shutter based on an optical fiber switch which is optically powered to provide a reference signal in an optical fiber pyrometer measuring from 200 to 550 °C. The tests show the potential of remotely powering via optical means a 300 mW power-hungry optical switch at a distance of 100 m, avoiding any electromagnetic interference close to the measuring point.

## 1. Introduction

Temperature measurement in today’s industrial environment requires sensors that are able to measure a large range of temperatures at relatively high speeds in localized areas, especially in machining processes [1,2] or rotor engines [3]. In the first case, a non-rotary high-speed cutting tool removes material from a rotary mandrel, and this rotary movement applied to the mandrel is provided by a high-power electrical motor. Additionally, the electromagnetic interferences emitted by the electrical motor limit the use of electrical-based sensors, especially thermocouples, because of the high sensitivity of the readings to the electromagnetic fields [4]. On the other hand, the use of infrared cameras for high-speed measurements in this framework is also limited by their low sampling rate operation as well as positioning issues, which make them unsuitable for very localized measurements in difficult-to-access areas [5,6]. In contrast to these sensors, radiation pyrometers overcome the flaws of electrical sensors using a non-contact technique based on the well-known spectral emission pattern from a black body given by the Wien–Planck relation [7], becoming a good choice for measuring temperature in these difficult-to-access rotating areas. However, a critical issue in radiation pyrometers results from their trade-off performance between providing a suitable spatial resolution and collecting enough radiated light amount to measure a specific temperature range. This disadvantage can be solved by employing a fiber-optic pyrometer capable of viewing the machining surface directly with a fiber-optic probe, thus providing localized temperature measurements but with different spot sizes. Spot diameters greater than 1 mm [2,3,8], from 0.8 to 0.25 mm [9,10], or as small as 0.16 mm [11] at the region of interest, have been reported. Nevertheless, within this approach, it is sometimes necessary to employ discrete optical components, such as condenser lenses [3], which may be difficult to place due to space restrictions or, if they are placed far away from the measurement point, require a line of sight that is not always available. 

However, a still important problem of fiber-optic pyrometers yet to solve is the difficulty of measuring low temperature values with both high accuracy and a moderate speed. This disadvantage limits their use when temperature measurement of polymer composites [12] or low-emissivity metals is required [13]. These materials are machined using lower speeds which generate lower radiation, thus reducing the temperature of the process. In this case, it is important to enhance the signal-to-noise ratio (SNR). Different pyrometers have been proposed in the low temperature range such as those based on pyroelectric detectors in the mid-infrared range to collect the higher energies radiated following the Wien’s displacement law but with limited accuracy and measurement speed. To overcome this issue, some solutions have been developed operating in the wavelength range from 1 to 4 μm and using infrared fibers with large core diameters [3,8,9,14] and cooling photodetectors. 

Other methods for measuring low radiated energy is to compensate for undesirable offsets in the receiving signal by applying a modulation scheme to the gathered light. This modulation can be performed using a shutter or a chopper to periodically interrupt the continuous light. This scheme is implemented for the measurement of the temperature distribution from 50 to 150 °C of large engines for power generation [3]. In other cases, the chopper provides the synchronous signal required in a lock-in amplifier, as in infrared multispectral optical fiber pyrometers wherein the influence of the reflected light by the hot body is taken into account thus providing a measuring range from 45 to 75 °C [9]. Nevertheless, temperatures in the whole range from 50 to 150 °C cannot be measured without the use of large diameter fibers, collimation lenses, different transimpedance amplifier gains, and active cooling systems. These two approaches offer good accuracy at low temperature values but at the expense of adding more complexity to the system, thus increasing its cost. There are other pyrometer applications where some sort of optical modulation is used. Particularly, a rotating shutter and specific data acquisition circuitry is proposed for measuring a broadband temperature range from 900 to 1200 °C [15]. The authors in [16] proposed using a modulator as a chopper, next to the hot object, to reduce the effect of measurement drifts due to background radiation on low temperature measurements (<200 °C), but they need an additional fiber, a mechanical chopper, and a CW Nd:YAG laser beam of 1 W to excite the modulator. However, the above solutions suffer from the serious drawback of the strong electromagnetic interference environment, which may interfere with the motor-driving of the mechanical chopper employed, thus affecting the uncertainty and synchronization of the readings, especially if a high accurate and at a moderate speed measurement is targeted. In contrast, a power over fiber (PoF) technique provides galvanic insulation between two ends of the fiber and a lack of electromagnetic interference among others. This is useful in current monitoring in high voltage transmission lines [17], in sensor networks in hazardous environments [18], or in providing an isolated power supply to a gate-drive [19], thus eliminating the control signal distortion in switching transients. For all these reasons, we consider PoF a good candidate for powering active parts of any fiber-optic pyrometer designs.

In this paper, we propose an improved design of a fiber-optic two-color pyrometer using InGaAs photodetectors with low-noise and single high-gain amplifiers to measure temperatures ranging from 170 to 530 °C in very localized areas and at a high speed. In another setup, for the first time to our knowledge, a light chopper based on an optically powered fiber-optic switch is used to provide a reference signal to reduce the effect of measurements drifts caused by the background radiation. The PoF technique becomes of prime importance to place the switch close to the hot object, thus minimizing the influence of additional energy radiation components and allowing for the use of a single chopper for both channels in the two-color pyrometer. The proposed topology also reduces the number of components within the system, thus avoiding synchronization issues especially if a multi-channel solution is considered. The use of a fiber-optic switch instead of a mechanical chopper also reduces the insertion losses and can enhance the SNR, critical in this noisy electromagnetic environment. The characteristics of the new elements at the selected spectral bands of operation are analyzed and the remote optically powering of the switch is discussed. Finally, calibration curves of both pyrometer prototypes with and without the fiber-optic switch are provided. 

## 2. Principle of Operation and Pyrometer Design

The spectral radiance of a blackbody is given by the well-known Planck’s law. If a real body with an emissivity ε is considered, the spectral radiance (*L*) can be expressed as [1]

(1) $$L\left(\lambda ,T\right)=\;\frac{C_{1}\cdot \varepsilon \left(\lambda ,T\right)}{\lambda^{5}\cdot \left(e^{\frac{C_{2}}{\lambda \cdot T}}\right)}$$

where *λ* is the wavelength, and *C*_1_ and *C*_2_ are the Planck’s radiation constants, whose values are 1.191 × 10^8^ W·Sr^−1^·μm^4^·m^−2^ and 1.439 × 10^4^ μm·K, respectively. *T* corresponds to the absolute temperature of the body.

In some cases, in order to overcome the influence of emissivity variations, two color fiber-optic pyrometers measuring the emitted radiation at two specific wavelength bands were used [1,2,8]. 

### 2.1. Pyrometer Design

**Figure 1:**
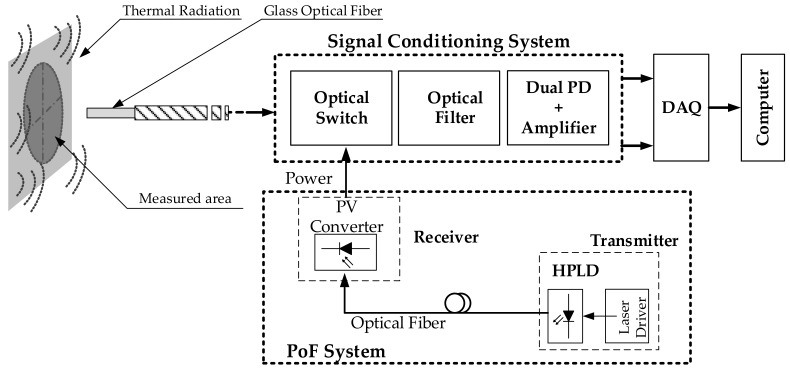
Schematic of the pyrometer with an optically powered fiber-optic switch. PD: photodetector; PoF: power over fiber; PV: photovoltaic; HPLD: high power laser diode. DAQ: data acquisition card.

The new pyrometer design is illustrated in Figure 1. It includes the fiber-optic switch, the optical fiber, and both photodetectors with their corresponding transimpedance amplifiers, thus performing the signal conditioning. The active element close to the measuring unit corresponds to the fiber-optic switch. It is optically powered by the PoF system and driven by an electrical square signal to perform an on/off control. Whether the optical switch will be part of the signal conditioning system depends on the pyrometer analysis to be performed. As in [1], the new pyrometer employs a 62.5/125 μm core/cladding diameter silica multimode optical fiber, thus allowing very localized temperature measurements without the need for condenser lens. The spot diameter provided by our solution at the region of interest results in 0.16 mm that matches with the fiber numerical aperture of 0.275 and a distance between the fiber end and the hot object of 0.3 mm. The signal processing system (Figure 1) is designed to be able to modulate the optical signal (on/off control) and to spatially demultiplex the radiation emitted by the hot object into two spectral bands centered at 1.31 and 1.55 μm, respectively. Two InGaAs photodetectors with high-gain transimpedance amplifiers are used to convert the receiving light from radiation to an output voltage. The optical switch is placed close to the hot object and before the optical filter for switching both spectral bands simultaneously.

### 2.2. Calibration Setup

**Figure 2:**
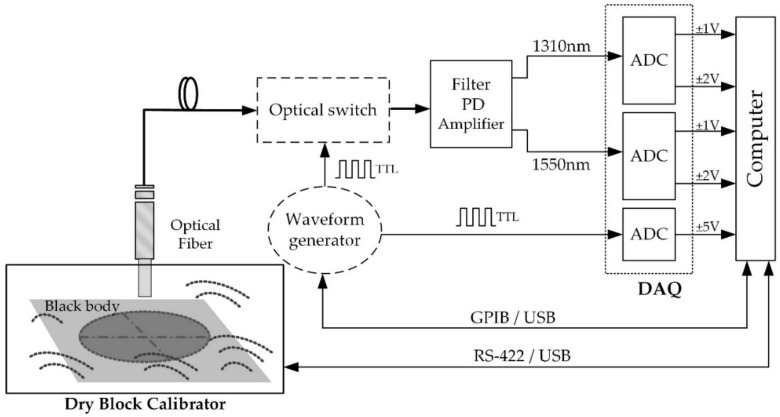
Schematic of the setup for the pyrometer calibration. Dashed lines are parts only included if the optical switch is considered. ADC: analog-to-digital converter.

An automated calibration setup is developed, as shown in Figure 2. This setup includes a data acquisition card (DAQ) with synchronous analog-to-digital converters (ADCs) for digitalizing the output voltage from the transimpedance amplifiers. The data acquisition card is configured to measure both voltages at two different voltage ranges (±1 and ±2 V). This feature is implemented to optimize the dynamic and voltage range of the ADCs with respect to the output voltage of the pyrometer. In addition, the optical switch control signal (TTL) is also acquired for synchronizing the voltage measurements with the fiber-optic switch.

The two-color pyrometer calibration is performed with a dry block calibrator and a black body kit as in [1,11]. The calibration setup operates within a temperature range from 50 to 650 °C with ±0.17 °C of temperature uncertainty (upper bound). Calibration results of the radiant flux emitted by the black body at 1.31 and 1.55 μm spectral bands, respectively, are measured placing the fiber sensor 0.3 mm from the reference blackbody by means of a calibrated metallic holder. The temperature span of the black body surface changes from 150 to 550 °C in 20 °C steps. Temperature acquisition is performed at a sampling rate of 1 kHz and taking 500 samples per temperature. A 45 min time interval between each temperature value acquisition in the calibration procedure is considered thus maximizing the stability of the measurements. Data processing computes on each step the average of the measured output voltage at both spectral bands from the mean of the samples acquired. 

Temperature measurements obtained from this calibration procedure are shown in the following section. The uncertainty of measured voltage according to [20] is the experimental standard deviation of the mean ($s\left(\bar{V}\right)$), defined as

(2) $$s\left(\bar{V}\right)=\sqrt{\frac{s^{2}\left(V_{k}\right)}{n}}$$

with

(3) $$s^{2}\left(V_{k}\right)=\frac{1}{n-1}\cdot \overset{n}{\underset{j=1}{\sum }}{\left(V_{j}-\bar{V}\right)}^{2}$$

where $V_{j}$ is the value of the output voltage in each observation, $\bar{V}$ is the average of all measurements, n is the number of independent repetitions or samples, $s\left(\bar{V}\right)$ is the experimental standard deviation of the mean, and $s^{2}\left(V_{k}\right)$ is the experimental variance of the observations.

On previous pyrometer designs, the pyrometer calibration curve was also validated using a K-type thermocouple [21].

## 3. Experimental Results

In this section, the characterization of some elements of the signal conditioning system and the measurements of the pyrometer calibration curves are provided, either with or without the fiber-optic switch. 

### 3.1. Two Color Fiber-Optic Pyrometer

**Figure 3:**
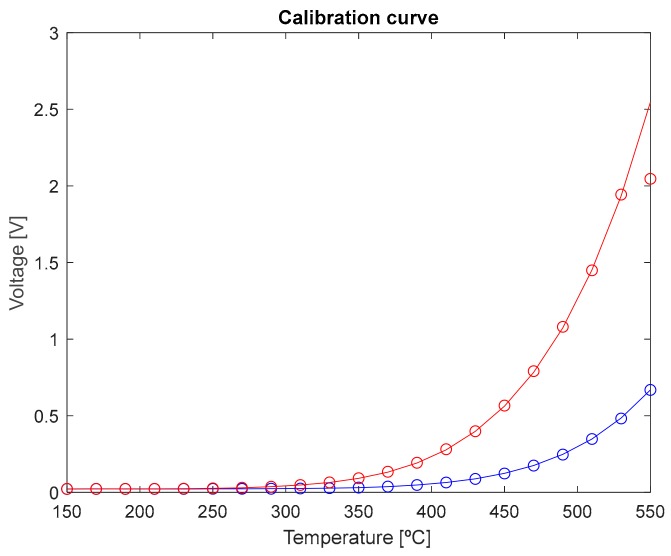
Experimental (○) and fitting (-) pyrometer calibration curves at (blue line) 1.31 μm and (red line) 1.55 μm, respectively. No fiber-optic switch is included within the setup.

Measurements average of the radiant flux emitted by the black body at both spectral bands of interest are shown in Figure 3. In those measurements, the temperature of the blackbody surface changes from 150 to 550 °C at 20 °C steps as described in the calibration setup section.

As expected the output voltage, which is proportional to the optical power gathered by the optical fiber, increases with temperature. In addition, the longer the wavelength band is, the greater the optical power measured. A single amplifier gain is used in the whole temperature range. The voltage measurements up to 530 °C are within the ±2 V upper limit operation range configured for the data acquisition card. This is why the experimental data at 550 °C does not fit to the calibration curve. The radiated energy by the hot object provides a voltage at the receiver greater than 2 V and the output of the DAQ is saturated. 

The experimental standard deviation of the mean, obtained from Equation (2), is almost constant (0.032 mV at 1.31 µm and 0.029 mV at 1.55 µm) being dominated by the dark and thermal noise of the receiver. To obtain the temperature error, the equivalence in temperature of the standard deviation of the mean of voltage measurements needs to be established. To do this, the following procedure was followed. First, the data were fitted to a five-order polynomial curve to obtain the relationship between voltage, $\bar{\;V}$, and temperature. Next, the standard deviation of the voltage mean value, $\;s\left(\bar{V}\right)\;$, was included in order to consider the case where the maximum error in the measurement occurs, as $V^{\prime }=\bar{V}+s\left(\bar{V}\right).$ Then, by solving the polynomial fitting, the temperature value corresponding to this voltage was calculated. Finally, the temperature error is given by

(4) $$E_{T}\left[\%\right]=\frac{T_{R}-T}{T}\cdot 100$$

where $T_{R}$ is the temperature corresponding to V′, and T is the temperature calibration.

After this analysis, it was verified that temperature uncertainties below 5% are obtained at both wavelength bands at temperatures greater than 190 °C. Particularly, if the 1.55 μm channel data is considered, temperature uncertainties are below 4%, even less than 1% for temperatures starting at 190 °C.

In addition, the calibration curve using only 150 samples instead of 500 samples is analyzed. Since the number of samples is smaller, the standard deviation of the mean increases slightly. However, this hardly influences the temperature uncertainty and in the voltage average. These values are almost identical to those obtained in the previous analysis. From this, it is concluded that reliable measurements could be obtained in this smaller time interval.

### 3.2. Two-Color Fiber-Optic Pyrometer with Fiber-Optic Switch

#### 3.2.1. Optical Switch: Characterization and Remote Power Over Fiber Tests

The pyrometer temperature error is not only dependent on the selected wavelength bands [2] but also on the spectral characteristics of the devices employed [1,11]. Consequently, it is important to characterize any new element being part of the pyrometer setup at both wavelength ranges. The 1 × 1 quad fiber-optic switch has 4 input ports and 4 output ports, which are simultaneously connected or disconnected by means of a 5 V electronic control signal. All switch ports are characterized although only Input Port 1 and Output Port 1 are used in the experiments. The following experimental data provided refers to these switch ports. Insertion losses less than 0.25 dB and crosstalk greater than 46 dB were measured at both spectral bands. The switching time, defined as the time elapsed to change the port state from off to 90% of the final value, was 1.55 ms with a standard deviation of 0.11 ms. The duty cycle of the output optical signal was around 60% when a 50% of duty cycle square pulse was used to control the switch, because the switch starts to change the state at a control signal slightly lower than specified. 

**Figure 4:**
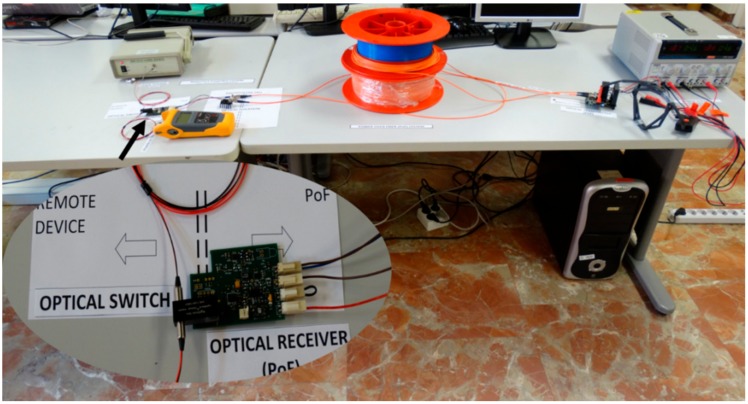
Picture of the 100-m-long Power over Fiber system at the laboratory. Inset: remote optically powered switch.

As previously stated, in case of operating the pyrometer in electromagnetic noisy environments such as rotor engines or machining equipment, Power over Fiber (PoF) can be a good technique for remotely powering the optical switch used as a chopper placed close to the hot object within the pyrometer sensing scheme. The optical switch is in front of the optical filter for switching both spectral bands simultaneously before they are spatially demultiplexed and detected by the corresponding photodetectors. The maximum power consumption of the fiber-optic is 300 mW (5 V × 60 mA). The PoF system deployed uses a transmitter made of 2 high power laser diodes (HPLDs) at 808 nm providing a total optical power of 3 W, two multimode optical fibers with a 200/500 µm core/cladding diameter, and a receiver that includes two GaAs optical photovoltaic (PV) cells, with on/off control capability via software. The maximum test distance explored for remote optically powering is 100 m, but shorter distance can be used with less power consumption. The optical fiber used in the pyrometer fiber-optic probe has low losses of around 0.3 dB/km, so a small resolution penalty should be expected if measurement must take place far away from the reception circuit. More specifications about the PoF system design are addressed in [18,22,23]. A picture of the PoF system that drives the fiber-optic switch is shown in Figure 4. 

#### 3.2.2. Calibration Curves of the Pyrometer with the Fiber-Optic Switch 

Measurements of radiant flux emitted by the blackbody using the pyrometer with the fiber-optic switch were carried out. The latter was excited by a square signal with a 50% duty cycle and a 10 Hz period. The temperature range, temperature steps, a sampling rate, and the number of samples were similar to that described in the calibration setup section. The uncertainty obtained at both wavelength ranges depended on the way the data was processed. Two data processing techniques were considered, and the samples of the two acquired periods were taken into account. In the first analysis, the output voltage amplitude average was calculated with the data corresponding to the on state of the switch (that is when the optical switch allows the radiant flow emitted by the black body to pass). The first 15 samples were excluded to avoid the influence of the delay time of the switch. The second technique was based on subtracting the voltage average of the on and off state of the switch (without taking into account the first 15 samples of each state). In this last method, since the final measure was obtained indirectly, the uncertainty was the combined standard uncertainty. 

**Figure 5:**
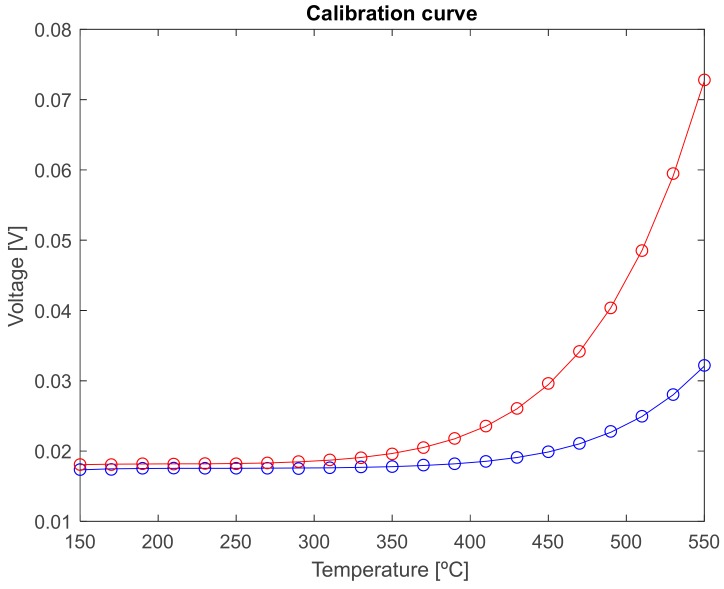
Experimental (○) and fitting (-) pyrometer calibration curves with the fiber-optic switch: (blue line) 1.31 μm and (red line) 1.55 μm.

The temperature errors were calculated following the same procedure described in the previous section after fitting the data to a polynomial curve of order five, whose approximation error was less than 1%. Slightly higher uncertainties are obtained when the data of the two states are subtracted, so this method is currently rejected. In Figure 5, the results for the data of the on state of the reference signal are shown. For example, the uncertainty can reach values below 5% whether only the 1.55 μm channel data is considered from 250 °C and below 1% from 310 °C.

Whether it is possible to obtain good results using fewer samples was evaluated in the first case. In this case, only data of one period of the on state, after the first 15 samples were removed, were used. Therefore, the number of samples used was 85.

The standard deviations of the mean obtained in this experiment were higher than those obtained with two periods. Even so, the difference in the temperature error was less than 1% and the voltage average was almost identical. It was concluded that, by acquiring the signal within a shorter period of time, reliable results could be obtained.

## 4. Discussion

The two-color fiber optic pyrometer using InGaAs photodetectors with low-noise and high-gain transimpedance amplifiers could measure temperature in very localized areas at a high speed and at a wider temperature range in comparison with our previous designs [1], particularly from 170 to 530 °C, with just a single gain factor. The electronic and optoelectronic devices used exhibited an excellent performance at frequencies up to 2 kHz. This value guarantees enough bandwidth to measure all possible temperature changes that happens during a machining process. To validate these measuring conditions, the previous sensing scheme was tested in a real application, which yielded excellent results in terms of response and accuracy [11].

Integrating a fiber-optic switch provided a reference signal but at the expense of having low level voltage signals at the pyrometer output. The difference in the output voltage amplitude with and without the switch increased at greater temperature values. For a 300 °C temperature value, the measured pyrometer sensitivity changed from around mV/°C to tenths of μV/°C. The main reason, apart from the additional fiber-optic switch insertion loss, arises from its 100 nm reduced bandwidth of at both spectral bands (1.26–1.36 µm and 1.51–1.62 µm, respectively). This affects the minimum temperature that can be measured with this pyrometer approach without using a lock-in amplifier, but prevents saturation at greater temperature values. The use of a broadband fiber-optic switch instead of a dual band counterpart will provide greater output voltages for the same temperature ranges. If measurements at a higher speed are required, other faster optical switch technologies can be used.

The proposed technique with the fiber-optic switch is an alternative in any of the different state-of-the-art pyrometer solutions previously described that take advantage of mechanical shutters, choppers, or lock-in amplifiers to provide background noise reduction, as part of a multispectral pyrometer or with the aim of avoiding excessive photodetector heating. Additional analysis of the fiber-optic switch performance in terms of jitter, operation frequency, and data processing is part of future work to perform these applications with lock-in amplifiers to recover the signal. 

The potential of optically powering the switch with a moderate power consumption at a large distance is also an attractive feature when measurements are carried out in electromagnetic noisy environments, such as engines, where electromagnetic interferences should be avoided to prevent interfere with the motor-driving of the mechanical chopper, thus affecting the uncertainty and synchronization of the readings.

The optical fiber coating was removed in the last section of the fiber-optic pyrometer probe, which was made of silica, which has a melting point close to 1400 °C. This temperature is two times higher than the maximum temperature reached in the calibration setup (650 °C). Therefore, the gathered properties and physical integrity of the glass fiber were not affected by the calibration temperatures as well as the cutting temperatures of the superalloys (up to 675 °C), at which the pyrometer was tested. 

Further analysis of pyrometer calibration under cooling and heating cycles should be performed in the future. Linearization of the ratio between both channels can be performed in a limited temperature range of around 100 °C.

## 5. Conclusions

An infrared two-color pyrometer able to measure temperature from 170 to 530 °C in very localized areas at a high speed with the same gain factor in the whole temperature range is reported. A light chopper based on a fiber-optic switch was tested to provide a 10 Hz reference signal within an optical fiber pyrometer that was able to measure at a moderate speed within a temperature range from 200 to 550 °C with proper data processing. A remote 300 mW power-hungry switch was optically powered at 100 m via a Power over Fiber system operating at 808 nm that employed a multimode fiber and GaAs photovoltaic cells. This Power over Fiber provided galvanic isolation and avoided any electromagnetic interference in the target applications ruled by electromagnetic noisy environments. It also allowed for placement of the optical switch in front of the hot object in case a reduction of the background noise is required.
